# Progress and Prospects for Titanium Extraction from Titanium-Bearing Blast Furnace Slag

**DOI:** 10.3390/ma17246291

**Published:** 2024-12-23

**Authors:** Yuxuan Qu, Lei Xing, Minglei Gao, Suxing Zhao, Qianqian Ren, Lanjie Li, Yue Long

**Affiliations:** 1 School of Metallurgy and Energy Engineering, North China University of Science and Technology, Tangshan 063000, China; yuxuanqu1011@163.com (Y.Q.); xingxi@stu.ncst.edu.cn (L.X.); 2 HBIS Material Technology Research Institute, Shijiazhuang 050023, China; gaominglei31135@hbisco.com (M.G.); zhaosuxing@hbisco.com (S.Z.)

**Keywords:** titanium-bearing blast furnace slag, direct utilization, titanium components, high-temperature carbothermic–low-temperature chlorination, integrated smelting

## Abstract

The composition of TBFS is complex. It is categorized into low (W_(TiO2)_ < 5%), medium (5% < W_(TiO2)_ < 20%), and high-titanium slag (W_(TiO2)_ > 20%) based on Ti content. The titanium in the slag is underutilized, causing it to accumulate and contribute to environmental pollution. Current methods for extracting titanium from TBFS include acid leaching, alkali fusion roasting, high-temperature carbonation–low-temperature chlorination, electrochemical molten salt electrolysis, and selective enrichment. However, these methods still face challenges such as environmental impact, high costs, low Ti recovery, and low Ti grade. This paper summarizes the mechanisms and characteristics of the above methods. Future research should focus on integrating pyrometallurgy with beneficiation processes, followed by further purification of titanium-rich phases through hydrometallurgy. Additionally, combining this with novel separation technologies (such as microwave and superconducting magnetic separation) will optimize the dissociation of titanium-bearing phases after enrichment.

## 1. Introduction

Titanium-bearing blast furnace slag (TBFS) is a byproduct obtained after the blast furnace smelting process [1]. It can be categorized into low (W_(TiO2)_ < 5%), medium (5% < W_(TiO2)_ < 20%), and high-titanium slag (W_(TiO2)_ > 20%) based on TiO_2_ content [2,3,4]. China generates approximately 7 million tons of TBFS annually, with accumulated storage exceeding 100 million tons. However, its utilization rate remains below 3% [5,6]. The large accumulation of slag not only occupies land resources but also causes pollution of nearby soils and water sources due to the heavy metals it contains [6]. At the same time, TBFS contains a significant amount of valuable metallic elements, especially titanium resources in medium and high-titanium slags. Given the increasing demand for titanium in current manufacturing industries, such as aerospace [7], equipment manufacturing [8], biomaterials [9], and catalytic industries [10] (Ti > 99%), the effective extraction of titanium from TBFS has become a key research focus [11], aiming to alleviate the shortage of titanium resources, address the large accumulation of slag, and reduce environmental pollution.

Currently, research on TBFS mainly focuses on medium- and high-titanium slags, with a low utilization rate of titanium resources in the slag. This is primarily due to the distribution of valuable titanium in multiple mineral phases [12], the high melting points of titanium-rich minerals [13], and the interlocking nature of these minerals [14], making it challenging to extract titanium from the slag. Several methods for extracting titanium from titanium slag have been developed, including acid leaching, alkali fusion roasting, and high-temperature selective enrichment [15,16,17]. However, various issues remain in industrial applications. For example, hydrometallurgy offers a high leaching rate (80–95%) [18,19,20,21] but involves high reagent concentrations, strong corrosiveness to equipment, and difficulties in treating waste acids. Pyrometallurgy processes large volumes with low pollution, but it suffers from low Ti recovery rates and high energy consumption. Thermodynamic phase diagrams, thermodynamic databases, and molecular dynamics have provided theoretical support for optimizing the thermodynamic conditions for titanium-rich phase enrichment, the movement of titanium-bearing phases in slags, and the transformation and separation of titanium-bearing phases [22,23,24].

Although progress has been made in extracting titanium from TBFS, there is a pressing need to develop a green, efficient, and large-scale industrial technology. This paper summarizes recent progress in titanium extraction, elaborates on the principles and characteristics of various methods, and presents prospects for integrating pyrometallurgy with hydrometallurgy.

## 2. Properties and Classification of Titanium-Bearing Blast Furnace Slag

**Figure 1:**
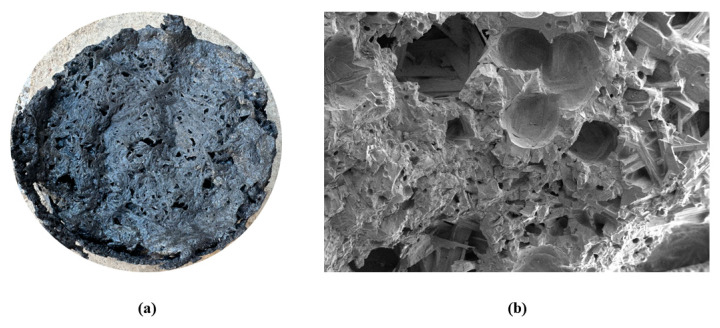
(**a**) Physical image of titanium slag; (**b**) SEM image of titanium slag.

TBFS is grayish-black in color, with a blocky and porous foam-like structure (Figure 1). It is classified into water-quenched slag and naturally cooled slag based on different cooling methods [25]. Water-quenched slag, due to rapid cooling, exhibits a dispersed structure, porosity, and relatively small particle sizes, with perovskite as the main phase. In contrast, naturally cooled slag cools more slowly, allowing sufficient time for phase growth, resulting in larger particles, with main phases including perovskite, titanaugite, and spinel [26,27].

**Figure 2:**
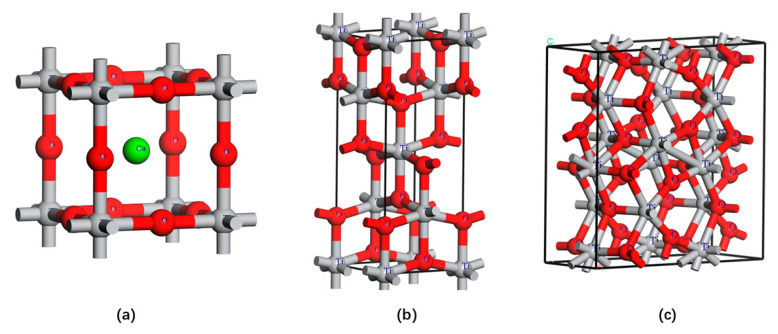
Crystal structures of titanium-bearing phases. (**a**) perovskite; (**b**) rutile; (**c**) anosovite.

Table 1 presents the chemical compositions of titanium slag from different regions in China [28,29,30], primarily including CaO, TiO_2_, MgO, SiO_2_, Al_2_O_3_, etc. The titanium component is mainly distributed in fine particles [31] within various mineral phases such as perovskite, titanium carbonitride, anosovite, rutile, pseudobrookite, and anatase. Table 2 lists the main titanium-bearing phases and their properties. The structure of titanium salt minerals in TBFS is influenced by the cooling rate. When the cooling rate is fast (water-quenched slag), most of the minerals take on a cross-shaped or dendritic structure; when the cooling rate is slow (naturally cooled slag), they exhibit a semi-automorphic structure [25]. Figure 2a,b shows the crystal structures of perovskite and rutile in titanium salts, respectively, while (c) shows the crystal structure of ilmenite. In (a), Ca^2+^ occupies the center of the cubic unit cell, forming a CaO_12_ cubic octahedron with 12 equivalent O^2−^ ions, while Ti occupies the corners of the cubic unit cell, forming a TiO_6_ cubic octahedron with 6 equivalent O^2−^ ions. In (b), Ti^4+^ ions occupy octahedral voids in the lattice, with each Ti^4+^ ion surrounded by 6 equivalent O^2−^ ions, forming a TiO_6_ octahedron. O^2−^ ions are arranged in a hexagonal close-packed manner, with each O^2−^ ion bonding with 3 equivalent Ti^4+^ ions. In (c), ilmenite crystallizes in the orthorhombic cmcm space group. Ti^3+^ forms bonds with six O^2−^ atoms, resulting in a mixture of TiO_6_ octahedra. O^2−^ ions bond with both Ti^3+^ and Ti^4+^, forming a mixture of OTi_4_ trigonal pyramids.

## 3. Extraction of Titanium Phases from Titanium-Bearing Blast Furnace Slag

### 3.1. Hydrometallurgical Methods

Common acidic solvents used in hydrometallurgical processes include sulfuric acid [32], hydrochloric acid [33], and mixed acids [34]. The acid leaching method utilizes the differing solubility of components in acidic solutions to dissolve titanium compounds from TBFS, while other solid impurities are retained in the slag. Sulfuric acid is typically used for titanium slag with low iron content, while hydrochloric acid is used in the opposite case [35,36].

#### 3.1.1. Sulfuric Acid Method

**Figure 3:**
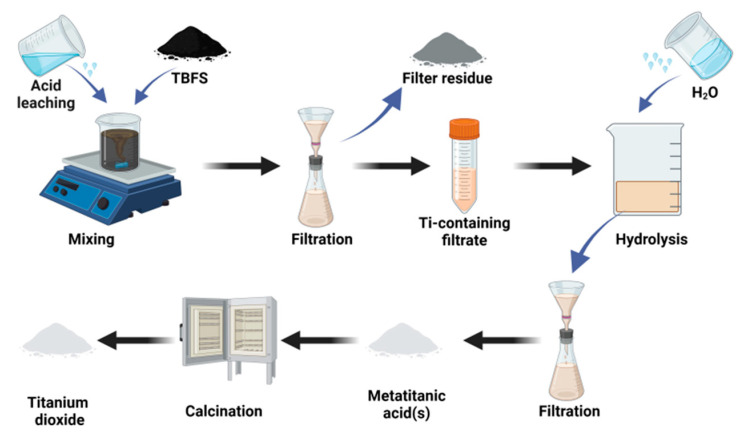
Flowchart for sulfuric acid leaching of titanium-bearing blast furnace slag.

The process flow for sulfuric acid leaching of TBFS is shown in Figure 3. Sulfuric acid is mixed with the slag, where MgO, Al_2_O_3_, and Fe_2_O_3_ react with sulfuric acid to form soluble sulfates, CaO reacts with sulfuric acid to form precipitated CaSO_4_, and TiO_2_ reacts with sulfuric acid to produce titanium sulfate solution (as shown in Equations (1)–(6)) [37]. The titanium sulfate solution is purified and then hydrolyzed to form the insoluble compound metatitanic acid (as shown in Equations (7) and (8)). After solid-liquid separation, metatitanic acid solid is obtained, followed by roasting to remove H_2_O, SO_2_, and other substances, ultimately yielding TiO_2_.

(1) $$\begin{array}{l} C\mathrm{aO}(s)+H_{2}SO_{4}(l)\to CaSO_{4}(s)+H_{2}O(l)\\ \Delta G(\mathrm{KJ}/\mathrm{mol})=-418.12-0.07T({}^{\circ}C) \end{array}$$

(2) $$\begin{array}{l} Al_{2}O_{3}(s)+3H_{2}SO_{4}(l)\to Al_{2}{\left({SO}_{4}\right)}_{3}(l)+3H_{2}O(l)\\ \Delta G(\mathrm{KJ}/\mathrm{mol})=-224.25+0.11T({}^{\circ}C) \end{array}$$

(3) $$TiO_{2}(s)+H_{2}SO_{4}(l)\to TiOSO_{4}(l)+H_{2}O(l)$$

(4) $$\begin{array}{l} MgO(s)+H_{2}SO_{4}(l)\to MgSO_{4}(l)+H_{2}O(l)\\ \Delta G(\mathrm{KJ}/\mathrm{mol})=-419.16+0.18T({}^{\circ}C) \end{array}$$

(5) $$Fe_{2}O_{3}(s)+3H_{2}SO_{4}(l)\to Fe_{2}{\left(SO_{4}\right)}_{3}(l)+3H_{2}O(l)$$

(6) $$F\mathrm{eO}(s)+H_{2}SO_{4}(l)\to FeSO_{4}(l)+H_{2}O(l)$$

(7) $$TiOSO_{4}(l)+2H_{2}O(l)\to H_{2}T{\mathrm{iO}}_{3}(s)+H_{2}SO_{4}(l)$$

(8) $$H_{2}T{\mathrm{iO}}_{3}(s)\to TiO_{2}(s)+H_{2}O(g)$$

During sulfuric acid leaching of titanium slag, increasing the liquid-to-solid ratio and acid concentration facilitates titanium dissolution. However, the initial liquid-to-solid ratio should not be too large [38]. In acid leaching, the dissolution of silicon forms a gel, making it difficult to effectively separate Ti and Si. Therefore, some researchers have explored the use of flocculants [39] and high-concentration acids [15], but these methods face challenges such as high costs, environmental pollution, and operational complexity. Wang et al. [18] conducted low-temperature curing of titanium slag using a mixture of sulfuric acid and waste acid for 3 h (sulfuric acid:waste acid:titanium slag = 1:2:1). The heat released caused H^+^ to break down the silicate structure, forming filterable SiO_2_. The material was then subjected to 2.5% H_2_SO_4_ leaching, hydrolysis, and calcination, where SiO_2_ entered the filter residue. The leachate contained Ti, Mg, and Al, with extraction rates of 87.29%, 93.21%, and 81.73%, respectively, while the solubility of silicon was only 1.24%. To investigate the low acid leaching rate of low-grade titanium slag, Li et al. [19] compared the acid leaching effects of low-grade titanium slag and titanium slag with a Ti content of 74%. For low-grade titanium slag, rapid acid leaching forms an insoluble layer on the slag surface, hindering the reaction. By lowering the initiation temperature and acid concentration, the acid leaching rate was reduced, increasing the acid leaching rate of titanium slag from 78.98% to 94.31%.

The advantages of sulfuric acid leaching include high extraction rates, low cost, mature technology, industrial production capability, and effective recovery of other metal resources. However, there are issues such as high acid consumption, high sulfuric acid concentration, the inability to treat waste acid, and environmental pollution. Additionally, the calcium sulfate (CaSO_4_) formed by sulfuric acid and calcium reacts to form an insoluble solid, which may cover the titanium-bearing phase surface in the slag during leaching, hindering sulfuric acid diffusion and limiting further improvements in titanium recovery.

#### 3.1.2. Hydrochloric Acid Method

**Figure 4:**
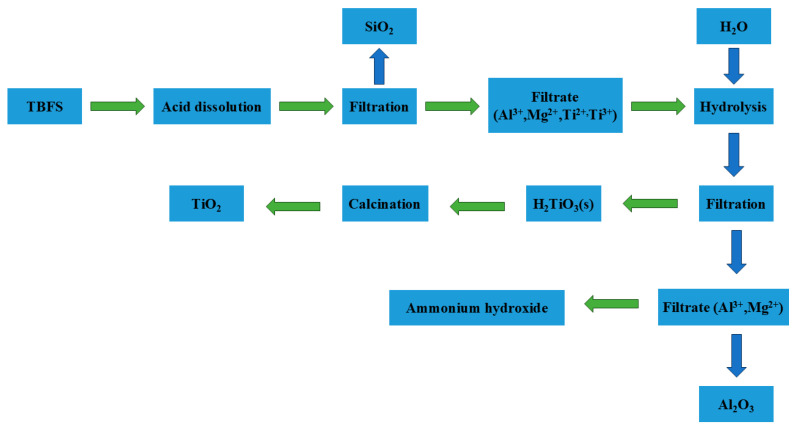
Flowchart for hydrochloric acid leaching of titanium slag.

In the process of leaching titanium slag with hydrochloric acid, the principle of hydrochloric acid leaching is similar to that of sulfuric acid leaching; however, hydrochloric acid exhibits a stronger ability to remove Fe. Since SiO_2_ is insoluble in hydrochloric acid during leaching, it exists in the form of filter residue, while other metal oxides that dissolve in hydrochloric acid exist as corresponding chlorides, as shown in Equations (11)–(14). Furthermore, reactions (9) and (10) allow for the removal of Fe metal from the leachate, followed by hydrolysis and calcination to obtain TiO_2_ (as shown in Equations (15)–(17)). The process flowchart is shown in Figure 4. Compared to sulfuric acid, the leaching rate of Ti is relatively lower. However, hydrochloric acid has a stronger ability to dissolve accompanying metals (Fe, Mg, Al), thus offering better impurity removal capacity.

(9) $$Fe(s)+2FeCl_{3}(l)\to 3FeCl_{2}(l)$$

(10) $$3FeCl_{2}(l)\to 3FeCl_{2}(s)$$

(11) $$TiO_{2}(s)+2HCl(l)\to TiOCl_{2}(l)+H_{2}O(l)$$

(12) $$CaO(s)+2HCl(l)\to Ca{Cl}_{2}\left(l\right)+H_{2}O(l)$$

(13) $$M\mathrm{gO}(s)+2HCl(l)\to MgCl_{2}(l)+H_{2}O(l)$$

(14) $$Al_{2}O_{3}(s)+6HCl(l)\to 2AlCl_{3}(l)+3H_{2}O(l)$$

(15) $$T\mathrm{iO}Cl_{2}(l)+3H_{2}O(l)\to H_{4}T{\mathrm{iO}}_{4}(s)+2HCl(l)$$

(16) $$H_{4}T{\mathrm{iO}}_{4}\to H_{2}T{\mathrm{iO}}_{3}(s)+H_{2}O(l)$$

(17) $$H_{2}T{\mathrm{iO}}_{3}(s)\to T{\mathrm{iO}}_{2}(s)+H_{2}O(g)$$

Gong et al. [40] used hydrochloric acid with a concentration of 5 mol/L to leach air-cooled high-titanium slag. The oxides of calcium, magnesium, aluminum, and iron form easily soluble chlorides, while titanium and silicon remain in the slag in the form of H_2_TiO_3_ and H_4_SiO_4_. Silicon was then removed through NaOH treatment, ultimately yielding titanium-rich material with 73% TiO_2_ content. Huang et al. [41] optimized the parameters for hydrochloric acid leaching of water-quenched titanium slag. Under conditions of HCl concentration at 33%, liquid-to-solid ratio of 15:1 (mL: g), reaction time of 30 min, and reaction temperature of 90 °C, the titanium leaching rate reached 75.3%. Hydrolysis and calcination of the leachate yielded 97.7% rutile TiO_2_, with the liquid-to-solid ratio and acid concentration having significant effects on titanium leaching. These studies optimized the parameters for HCl leaching of water-quenched and air-cooled slags, improving the titanium recovery rate.

Compared to sulfuric acid, hydrochloric acid is more effective at removing accompanying metals and can efficiently remove iron oxides, though it results in a lower titanium leaching rate. Since hydrochloric acid leaching does not form insoluble solids like calcium sulfate (CaSO_4_), the treatment of solid waste is relatively simple. However, hydrochloric acid is volatile and, if not managed properly, may lead to acid mist pollution. Additionally, its strong corrosiveness places higher demands on equipment.

#### 3.1.3. Mixed-Acid Method

**Figure 5:**
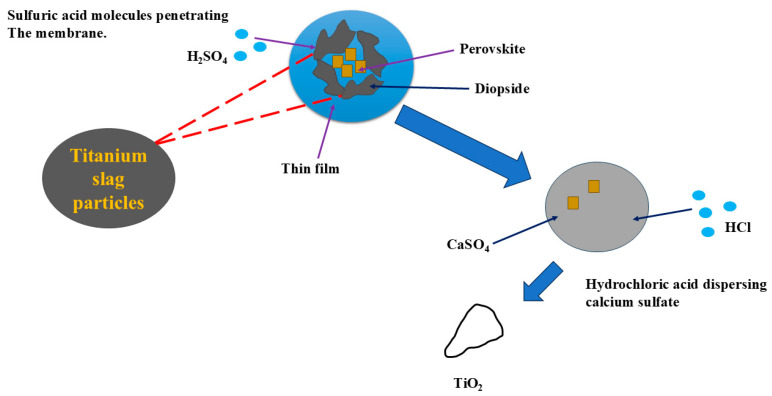
Schematic of mixed-acid leaching of TBFS.

The main mineral phase of air-cooled titanium slag, perovskite, is embedded in the diopside. During the early stages of acid leaching, the acid first reacts with diopside to form aluminosilicates and silica gels, creating a thin film on the surface of titanium slag particles. Sulfuric acid molecules can pass through this film and erode the diopside on the surface of the perovskite, allowing the acid to react with the perovskite. During the reaction, the generated CaSO_4_ hinders further reaction, but hydrochloric acid can disperse CaSO_4_, facilitating continued reaction. The process flowchart is shown in Figure 5. Li et al. [42] used a mixed acid with a sulfuric acid to hydrochloric acid volume ratio of 3:1 and a concentration of 6 mol/L. The main components in the filter residue were CaTiO_3_, CaSO_4_, and metatitanic acid, with a TiO_2_ content of up to 30%. Acid-soluble components such as Mg, Al, and Ca entered the filtrate, with a CaO leaching rate of 64%, MgO leaching rate of 88%, and Al_2_O_3_ leaching rate of 86%. However, no further treatment was carried out on the filter residue.

Tian et al. [34] optimized the leaching effect using a sulfuric acid-chloric acid mixed solution with a Cl^−^ to SO_4_^2−^ molar ratio of 1:1. They reacted the complex, single-acid-insoluble perovskite-type titanium slag (W_TiO2_ = 40%) with the solution at 65 °C for 10 h to obtain titanium oxy-sulfur-chloride solution and silica residue. The hydrolysis was then carried out at 160 °C for 4 h, with a hydrolysis rate exceeding 90%. Finally, metatitanic acid was obtained after filtration, and the titanium leaching rate exceeded 90%.

This method achieves the leaching of insoluble perovskite, recycles both acid and water and avoids the formation of solid waste (CaSO_4_), resulting in lower energy consumption. The mixed acid leaching method is suitable for processing complex mineral compositions. Compared to single-acid leaching, this method combines the characteristics of multiple acids, enabling more comprehensive leaching of titanium slag titanium and other metal elements, significantly improving titanium yield. Sulfuric acid effectively dissolves titanium from perovskite, while hydrochloric acid further processes other metal oxides. When titanates are difficult to dissolve, mixed acids demonstrate a stronger leaching ability. However, this process is relatively complex, costly, and requires large amounts of acid, posing significant environmental pollution risks. Precise control of the acid ratio and reaction conditions is also necessary.

#### 3.1.4. Alkali Fusion and Salt Roasting

**Figure 6:**
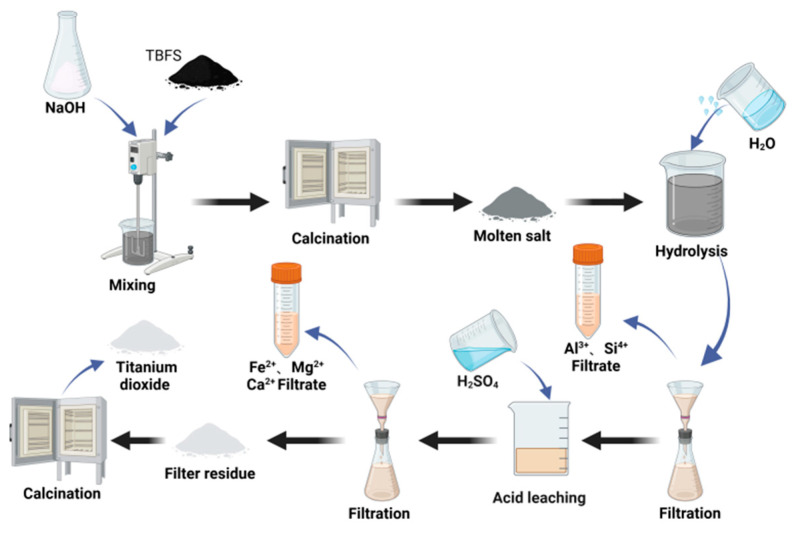
Process flowchart for TiO_2_ production through alkali fusion and salt roasting.

In the extraction of Ti from TBFS, the main impurities include MgO, Al_2_O_3_, Fe_2_O_3_, SiO_2_, and CaO. In Figure 6, since TiO_2_ (rutile) has almost no reaction with alkaline reagents, Si and Al metal oxides react with alkaline oxides to form water-soluble salts (such as NaAlO_2_, Na_2_O·nSiO_2_), facilitating the separation of Si, Al, Na_2_TiO_3_, and other unreacted materials, and removing metal oxides that are difficult to leach with acid reagents. Ti enters the filter residue and further reacts with acid, allowing Na_2_TiO_3_ to separate from other oxides (including FeO, Fe_2_O_3_, MgO, etc.). This is followed by calcination to obtain TiO_2_. When using NaOH as an alkaline reagent, different titanium phases react with NaOH at different temperatures. For example, the reaction temperature for the anosovite phase is >1200 °C, while for the perovskite phase it is <600 °C. To further reduce the reaction temperature, Sun et al. [43] used Na_2_CO_3_ as the alkaline reagent. The reaction can be carried out at 1000 °C, achieving a titanium extraction rate of up to 80%. Some studies have shown that under high pressure, reacting titanium-rich titanium slag with alkaline solution to remove impurities results in higher titanium extraction rates and lower reaction temperatures. Relevant studies are listed in Table 3 below.

Alkaline molten salt calcination increases the leaching rate and is simple to operate, but the rapid pH change results in significant consumption of acids and alkalis, leading to equipment corrosion. When NaNO_3_ and Na_2_CO_3_ are used as alkaline reagents, care must be taken to prevent the generation of harmful gases such as CO and NO.

(18) $$SiO_{2}(s)+2NaOH(s)\to Na_{2}SiO_{3}(s)+H_{2}O(g)$$

(19) $$Al_{2}O_{3}(s)+2NaOH(s)\to 2NaAlO_{2}(s)+H_{2}O(g)$$

(20) $$TiO_{2}(s)+2NaOH(s)\to Na_{2}TiO_{3}(s)+H_{2}O(g)$$

(21) $$Na_{2}TiO_{3}(s)+2H_{2}O(l)\to H_{2}TiO_{3}(s)+2NaOH(s)$$

(22) $$Na_{2}TiO_{3}(s)+TiO_{2}(s)\to Na_{2}TiO_{3}(s)+2NaOH(s)$$

#### 3.1.5. Ammonium Sulfate Method

**Figure 7:**
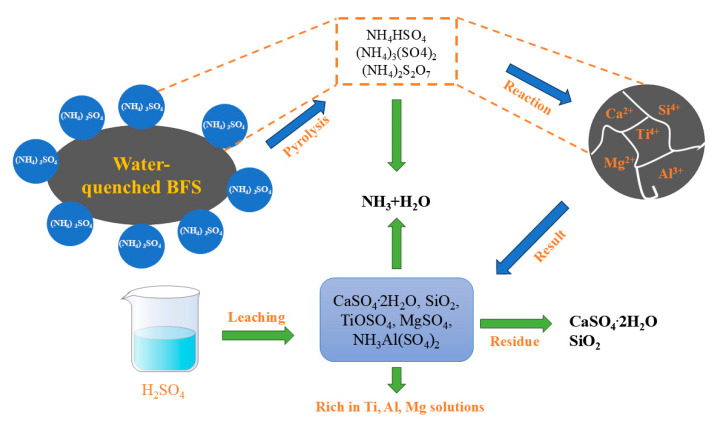
Process flowchart for ammonium sulfate pyrolysis and acid leaching.

The ammonium sulfate melting process for titanium extraction utilizes the characteristic thermal decomposition of ammonium sulfate at 205 °C [47,48]. At this temperature, gases such as CN_3_ and H_2_SO_4_ generated by ammonium sulfate affect the conversion rates of metals like Mg, Al, and Ti, necessitating operation under pressurized conditions. The process consists of three main steps (Figure 7): pressurized pyrolysis, sulfuric acid leaching, and hydrolysis calcination. During the pyrolysis stage, intermediate products of ammonium sulfate decomposition (NH_4_HSO_4_, (NH_4_)_3_(SO_4_)_2_, and (NH_4_)_2_SO_7_) spontaneously react with mineral phases such as perovskite and spinel in the titanium slag, converting the slag into soluble metal sulfates (CaSO_4_, TiOSO_4_, MgSO_4_, and NH_4_Al(SO_4_)), as shown in Equations (23)–(27). These metal sulfates dissolve in sulfuric acid, allowing metal elements to enter the solution, which is then transformed into TiO_2_ through hydrolysis and calcination. Bian et al. [49] mixed ammonium sulfate with titanium slag and calcined the mixture at 370 °C for 90 min, followed by leaching at 90 °C, 450 rpm, and 10% H_2_SO_4_, and finally hydrolyzed the leachate under boiling conditions. The product contained 94.1% TiO_2_, and the residue could be treated by adjusting the pH and conducting multiple hydrolysis steps to recover metals such as Al and Mg.

(23) $${\left(NH_{4}\right)}_{2}{SO}_{4}\to {NH}_{4}HSO_{4}+{NH}_{3}$$

(24) $$2NH_{4}HSO_{4}+TiO_{2}\to {\mathrm{TiOSO}}_{4}+{\left(NH_{4}\right)}_{2}SO_{4}+H_{2}O$$

(25) $$2NH_{4}HSO_{4}+CaO\to {\mathrm{CaSO}}_{4}+{\left({NH}_{4}\right)}_{2}SO_{4}+H_{2}O$$

(26) $$2NH_{4}HSO_{4}+MgO\to {\mathrm{MgSO}}_{4}+{\left({NH}_{4}\right)}_{2}SO_{4}+H_{2}O$$

(27) $$6NH_{4}HSO_{4}+{Fe}_{2}O_{3}\to {\mathrm{Fe}}_{2}{\left({\mathrm{SO}}_{4}\right)}_{3}+3{\left(NH_{4}\right)}_{2}SO_{4}+3H_{2}O$$

The ratio of titanium slag to ammonium sulfate, reaction time, and reaction temperature have a significant impact on the titanium leaching rate [50]. This method not only enables the recycling of ammonium sulfate and ammonia gas and the effective separation of titanium but also allows for the further recovery of metals such as Al and Mg, achieving comprehensive resource utilization. However, the technology is not yet mature, the operation is difficult, the process is complex, and large-scale production remains challenging.

#### 3.1.6. Comparison and Analysis of Methods

Table 4 compares the characteristics and applicability of hydrometallurgical leaching technologies. The mixed acid method is suitable for complex, difficult-to-dissolve mineral phases and allows for the recovery of multiple metals, but it emits harmful gases. However, the titanium leaching rate is only 80%. Ammonium sulfate reacts with titanium to generate soluble sulfates, reducing the sulfuric acid concentration. The study focused on titanium slag with Ti content of 18–27%. The alkaline molten salt method is effective for titanium slags dominated by rutile. Using HCl acid leaching provides a high impurity removal capacity and indirectly increases the titanium recovery rate. However, for titanium slags dominated by perovskite, sodium–calcium silicates are formed during alkaline leaching, which reduces the titanium content. All of the above methods face challenges such as high costs, technological immaturity and process complexity. HCl leaching is suitable for titanium slags with low TiO_2_ content (<35%) and abundant associated metals. It has a simple process, with a leaching rate of 70–90%. Sulfuric acid leaching is the only mature and industrially viable technology, suitable for high Ti content titanium slags with few associated metals, achieving a leaching rate of 80–95%. Future research should focus on reducing acid concentration and improving waste acid treatment technologies.

### 3.2. High-Temperature Carbothermic and Low-Temperature Chlorination

**Figure 8:**
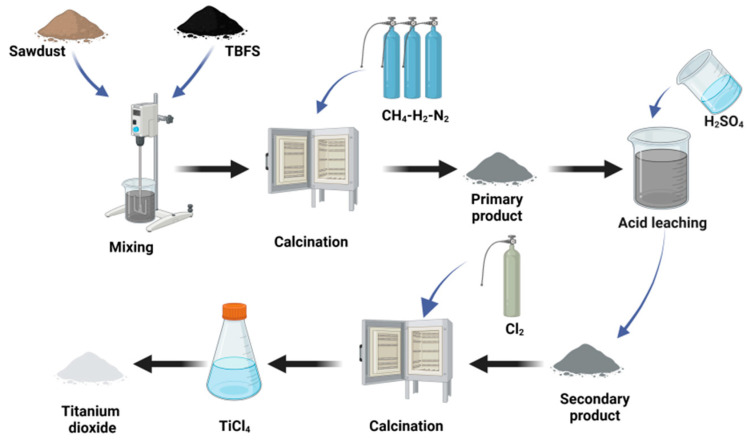
New process flowchart for CH_4_–H_2_–N_2_ mixed gas reduction and low-temperature chlorination.

High-temperature carbonization and low-temperature chlorination is a technique for producing TiCl_4_ from titanium slag, to use TiCl_4_ as a raw material for producing sponge titanium and titanium dioxide powder [54]. The process flow diagram is shown in Figure 8. The principle of this process is to react the slag with coke at high temperatures (>1600 °C) to obtain Ti(C, N). Since the chlorination rate of Ti(C, N) is much higher than that of other oxides, this method allows for the selective chlorination of Ti(C, N), thereby keeping impurities in the slag [55,56,57]. Chlorine gas (Cl_2_) is then introduced under appropriate temperature and atmospheric conditions, converting the product into chloride (TiCl_4_). This method efficiently recovers the valuable element titanium, but the high-temperature carbonization stage requires significant energy consumption and places high demands on refractory materials [58]. Fan et al. [6] treated complex titanium slag with an 8% CH_4_–84% H_2_–8% N_2_ mixed gas, lowering the temperature required for the reduction of TiN to Ti(C, N, O) and its chlorination [55,59]. The experiment was conducted at 1150 °C for 8 h, with 5% Fe_2_O_3_ and sawdust or urea added to promote the reaction and accelerate the gas–solid reaction. After reduction, HCl was used to remove Fe, Ca, and Mg oxides, and Cl_2_ was introduced to generate TiCl_4_. The Ti(C, N, O) content was 20.1%, with a Ti conversion rate of 96%. The process flow is shown in Figure 8. with the main reactions including:

(28) $$CH_{4}↔C+2H_{2}$$

(29) $$C+H_{2}O↔CO+H_{2}$$

(30) $$C+CO_{2}↔2CO$$

(31) $$CaTiO_{3}+3CH_{4}↔TiC+CaO+2CO+6H_{2}$$

(32) $$CaTiO_{3}+2CH_{4}+0.5N_{2}↔TiN+CaO+2CO+4H_{2}$$

High-temperature carbonization and low-temperature chlorination is the only method capable of achieving semi-industrial production. This method improves the extraction and enrichment rates of titanium components. However, the method currently faces the following issues: the high-temperature carbonization stage consumes large amounts of energy, low-temperature chlorination waste requires proper disposal methods, the reaction time is long, and the reactivity between slag and coke is poor. The use of CH_4_–H_2_–N_2_ mixed gas for reduction and carbon–nitrogen processing significantly reduces the issue of high energy consumption, demonstrating many potential advantages and feasibility. However, further solutions to problems such as impurity removal are needed in the future.

### 3.3. Electrochemical Molten Salt Electrolysis

**Figure 9:**
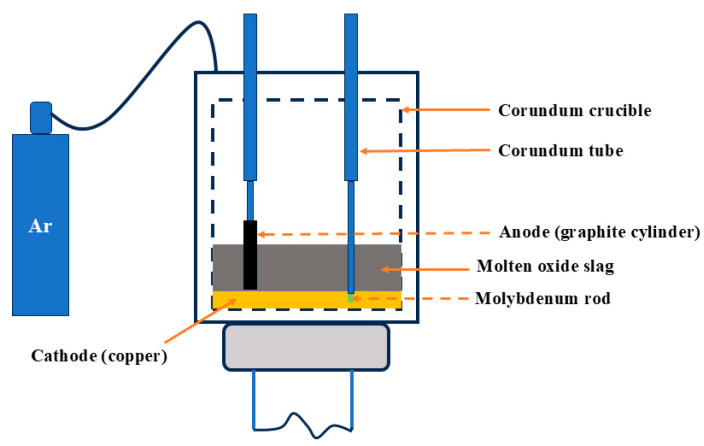
Process flowchart for electrochemical molten salt electrolysis.

Electrochemical molten salt electrolysis is an alternative titanium extraction method, with electrolytes including molten oxides [60] (CaO–MgO–SiO_2_–Al_2_O_3_–TiO_2_) and molten salts [61] (NaCl, CaCl_2_). In this process (Figure 9), liquid metal is used as the cathode, and graphite as the anode, with Ti ions being reduced at the cathode. Due to the favorable diffusion behavior of the liquid electrode, metal oxidation during deposition is avoided. Metals in the liquid cathode have lower activity, allowing metal ion precipitation potential to shift positively, resulting in depolarized deposition. The cathode metal and deposited metal are enriched as an alloy. Research shows that the decomposition voltages of molten oxides follow the order: MgO > CaO > Al_2_O_3_ > TiO_2_ > SiO_2_ [12]. When molten salts are used as the electrolyte, titanium slag acts as the cathode, producing metallic titanium through the electrodes. Marshall et al. [62] used iridium metal for both the cathode and anode in direct current electrolysis of molten CaO–MgO–SiO_2_–Al_2_O_3_–TiO_2_ electrolyte. The study showed that SiO_2_ is more easily reduced than TiO_2_, leading to the co-deposition of titanium and iridium. PU et al. [12] used liquid copper as the cathode. Due to the strong bonding between Ti and Cu, Ti is preferentially reduced, forming a Ti–Cu alloy. In low-titanium slags, Ti and Si undergo co-reduction, forming a Ti–Si alloy, which enables partial extraction of alloys.

Compared to other methods, electrochemical metallurgy is an efficient and environmentally friendly production process [63,64], with final enrichment in the form of alloys, enabling the utilization of secondary resources.

### 3.4. Ultragravity Metallurgy Process

**Figure 10:**
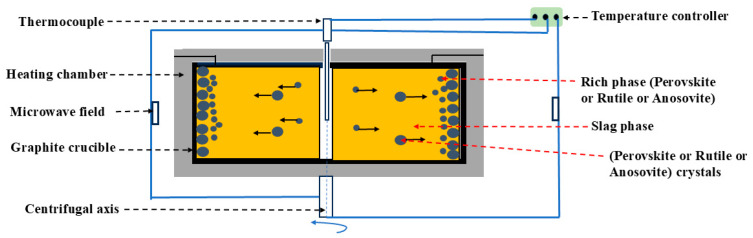
Process flowchart for ultragravity metallurgy.

A supergravity field is generated through a centrifugal field. As the gravity coefficient (Equation (33)) increases, the mass transfer between molecules and phases accelerates significantly, leading to larger grain diameters as the centrifugal rate of the grains increases [65]. In the supergravity field, by altering the physicochemical conditions of the slag, one of the titanium-containing phases is enriched towards rutile, perovskite, or ilmenite. The enriched phase then migrates directionally to the bottom of the liquid slag along the supergravity direction, while the liquid slag moves upwards in the opposite direction (Figure 10). The directional migration is primarily controlled by the driving force F (Equation (34)), buoyancy Fb (Equation (35)), and drag force F_d_ (Equation (36), with the drag coefficient related to viscosity (Equation (37)). Li et al. [66] found that at a gravity coefficient G = 750, t = 25 min, and T = 1305 °C, the TiO_2_ content in perovskite at the bottom of the interface was 34.65%, with a Ti recovery rate of 75.28%. Based on this, Gao et al. [67] used a filtration method, with crucibles placed in an upper–lower configuration and a carbon fiber layer in between for filtering. This allowed the liquid slag to separate from the solid perovskite in the supergravity field, flowing through the carbon fiber layer to the lower crucible. The TiO_2_ content in the perovskite was 46.36%, and the Ti recovery rate was 78.17%. Du et al. [65] determined the temperature range for the coexistence of solid and liquid rutile and used supergravity separation to obtain rutile with a TiO_2_ content of 95.56%.

(33) $$G=\frac{\pi^{2}N^{2}R}{900g}$$

(34) $$F=\frac{\pi }{6}{d^{3}}_{p}Gg\rho_{p}$$

(35) $$F_{b}=\frac{\pi }{6}{d^{3}}_{p}gG$$

(36) $$F_{d}=\xi \frac{\pi {d^{2}}_{p}}{4}\rho \frac{u^{2}}{2}$$

(37) $$\zeta =\frac{24\eta }{d_{p}u\rho }$$

Compared to conventional gravity, in supergravity conditions, the mass transfer rate between molecules and titanium-containing phases is significantly higher, allowing for the separation of enriched phases. However, due to the high viscosity of high-titanium slag, crystallization and separation parameters still require further study.

### 3.5. Metallothermic Reduction

The strong reducing properties of silicon and aluminum make them ideal reducing agents for the high-temperature reduction of titanium oxides. In this process, titanium is reduced from titanium oxides and forms titanium alloys with other metal elements (Al, Nb, Cr, Ni, etc.) [68], which have acid–alkali resistance and high elasticity [69]. Alloys are prepared from TBFS primarily through aluminothermic methods (such as Equations (38)–(41)) and silothermic methods (such as Equations (42)–(43)). Regardless of whether SiO_2_ and Al_2_O_3_ are generated during aluminothermic or silothermic reduction, the slag viscosity tends to be high, necessitating the addition of fluxing agents (CaO, Fe_2_O_3_) to lower the slag viscosity [70]. Table 5 summarizes the findings of researchers who used Si and Al as reducing agents, for reference by relevant personnel.

(38) $$3T{\mathrm{iO}}_{2}+4Al\to 2Al_{2}O_{3}+3Ti$$

(39) $${3\mathrm{SiO}}_{2}+4Al\to 2Al_{2}O_{3}+3Si$$

(40) $$3\mathrm{FeO}+2Al\to Al_{2}O_{3}+3Fe$$

(41) $${\mathrm{Fe}}_{2}O_{3}+2Al\to Al_{2}O_{3}+2Fe$$

(42) $$3T{\mathrm{iO}}_{2}+8\mathrm{Si}\to 3SiO_{2}+Ti_{3}Si_{5}$$

(43) $${\mathrm{CaTiO}}_{3}+\mathrm{Si}\to SiO_{2}+Ti+CaO$$

The metal thermal reduction method for treating TBFS results in a low Ti recovery rate, and the alloy formed after reduction and separation contains impurities such as Mn and Fe. Currently, there is limited research on this method compared to other methods, and future studies need to focus on impurity removal from alloys and improving Ti recovery rates.

### 3.6. Combination of Pyrometallurgical and Hydrometallurgical Methods

The combined pyrometallurgical and hydrometallurgical treatment method integrates the advantages of both processes. Pyrometallurgical metallurgy achieves large-scale preliminary enrichment of valuable titanium elements and removes impurities that are difficult to dissolve at high temperatures. This is followed by hydrometallurgical metallurgy to further purify the material, removing impurities such as Mg and Al, thereby yielding high-purity titanium products. Liu et al. [74] mixed titanium slag with 7.5% H_3_PO_4_ and roasted the mixture at 1000 °C for 2 h. The roasted sample was then leached with 20% and 40% H_2_SO_4_, resulting in 88.54% TiO_2_. During the pyrometallurgical stage, H_3_PO_4_ promotes the conversion of titanium oxides to the rutile phase and disrupts the silicate structure, making it easier for hydrometallurgy to remove impurities. In Safdara et al. [75], carbon reduction combined with weak acid leaching was used. First, 6% carbon was added and pretreated at 1000 °C for 3 h to obtain highly active TiC. The sample was then leached with 0.2 mol/L sulfuric acid at 80 °C and a liquid-to-solid ratio of 100:1. Most of the Fe and other elements were transferred into the solution, and the remaining residue primarily consisted of rutile with 72.2% TiO_2_.

This method is applicable for treating a variety of slags. Based on the characteristics of titanium slag, the treatment process can be optimized by adjusting the pyrometallurgical pre-treatment conditions and hydrometallurgical leaching parameters. During the pyrometallurgical pre-treatment stage, high-temperature treatment can break down part of the silicate structure and promote the enrichment of titanium-containing phases towards specific phases. This lays the foundation for subsequent hydrometallurgical treatment and reduces the concentration of acid reagents required.

### 3.7. Integrated Mineral Processing and Metallurgy

The integrated beneficiation and smelting process for titanium slag combines mineral processing and smelting technologies to effectively recover titanium (Ti). This process controls the cooling rate, alkalinity, and additives to enrich the dispersed titanium components in medium- to high-titanium slag into specified titanium-containing phases (such as rutile, anosovite (Mg, Fe) Ti_3_O_5_, and perovskite), thereby promoting the growth of the enriched phases. Due to differences in density, floatability, and other properties between the enriched titanium-containing phases and other minerals, physical separation techniques such as gravity separation, magnetic separation, and flotation are used to effectively separate titanium-rich anosovite from other impurities. The process flow diagram is shown in the figure.

#### 3.7.1. High-Temperature Selective Enrichment

A certain amount of CaO and Fe_2_O_3_ is added to the slag to adjust the initial amount of perovskite and reduce viscosity, thereby promoting ion migration and increasing the crystallization of perovskite (crystallisation rate: 30.62%; crystalline volume: 63.17 um [17]). The cooling rate and alkalinity have a significant impact on the crystallization of perovskite. Studies show that a lower cooling rate (0.5 °C/min) [17] and higher alkalinity (R > 1.2) are beneficial for the growth of perovskite [76]. At higher alkalinity, the mass fraction of CaO increases, the mass fraction of SiO_2_ decreases, and the concentration of Ca^2+^ and O^2−^ ions in the slag increases, further promoting the enrichment of perovskite. Low alkalinity favors the enrichment of anosovite and rutile [77,78]. However, during the enrichment process, silicates may encapsulate the titanium-containing phases [79]. The microwave method utilizes differences in microwave absorption, density, and melting points between perovskite and other components to detach the titanium-containing phases from silicates, thus facilitating the enrichment of low-valent titanium to high-valent titanium, with a recovery rate of 84.15% [80].

The enrichment of anosovite occurs in a reducing atmosphere. Adding 42–50% SiO_2_ to the slag, the Gibbs free energy of reactions (45) and (46) is smaller than that of reaction (44), causing CaO to react with SiO_2_ first, indirectly inhibiting reaction (44) [81]. Rutile serves as the enriched phase, with ZrO_2_ acting as a nucleating agent, promoting the formation of uniform rutile crystals, which is beneficial for subsequent beneficiation [82]. B_2_O_3_ and TiO_2_ accelerate the enrichment and recrystallization of rutile [83,84] (crystallisation rate: 21.83%; crystallisation volume: 200–300 um).

(44) $$CaO+TiO_{2}\to CaO•TiO_{2}$$

(45) $$CaO+2SiO_{2}+MgO\to CaMgSi_{2}O_{6}$$

(46) $$CaO+2SiO_{2}+Al_{2}O_{3}\to CaAl_{2}Si_{2}O_{8}$$

#### 3.7.2. Mineral Separation of Titanium-Containing Phases

Table 6 summarizes the mineral separation of titanium-containing phases, showing that single beneficiation techniques do not yield satisfactory results for separating titanium-containing phases. Due to the similar density of perovskite to that of pyroxene and the similar magnetic properties to rutile, it is difficult to separate it using magnetic separation and gravity separation [24]. Perovskite also shows differences in floatability from gangue minerals, which theoretically allows for its separation via flotation. While flotation can successfully separate perovskite in synthetic ores, its separation from titanium-rich blast furnace slag is not ideal, possibly due to the interlocking of phases within the high-titanium slag. The densities of ilmenite and rutile are 4.19 g/cm^3^ and 4.20 g/cm^3^, respectively, which show significant density differences compared to other minerals. Therefore, they can be effectively separated using gravity separation. The specific magnetization of rutile is 14.51 × 10^−6^ (cm^3^g^−1^), which enables the separation of magnetic minerals through magnetic separation. Integrated beneficiation techniques result in higher recovery rates compared to single beneficiation methods. However, improving the recovery of enriched phases through beneficiation after pyrometallurgical treatment remains a key focus for future research.

After the integrated beneficiation and pyrometallurgy process, further hydrometallurgical methods are employed to remove residual impurities (e.g., Mg, Al, and other metal oxides), achieving further purification. This integrated process significantly improves the recovery rate of titanium resources, optimizes the production process, and provides new technological pathways and ideas for the development of the titanium industry. Future research could focus on the formation mechanisms and crystal growth mechanisms of ilmenite phases, exploring optimal process parameters, such as controlling slag composition, redox conditions, and temperature regimes, to further improve titanium enrichment efficiency. Currently, there is limited research on the ilmenite phase. Similarly, during the beneficiation stage, novel separation techniques, such as microwave-assisted and superconducting magnetic separation, can be integrated. During the hydrometallurgical stage, environmentally friendly leaching processes, such as low-concentration acid systems and electrochemical-assisted leaching, need to be developed to reduce the difficulty of waste acid treatment and achieve green production. This method is a comprehensive approach for treating titanium slag, integrating hydrometallurgy, pyrometallurgy, and beneficiation processes. Compared to other methods, the integrated beneficiation and pyrometallurgy process offers higher titanium recovery and higher titanium grade, presenting significant advantages for future titanium slag processing.

## 4. Conclusions

This paper summarizes the basic principles, technological advantages, limitations, and applicability of methods such as pyrometallurgy (high-temperature carbothermic–low-temperature chlorination and high-temperature selective enrichment), hydrometallurgy (sulfuric acid, hydrochloric acid, mixed acid, and alkaline molten salt roasting methods), electrochemical processes, and hypergravity separation for treating titanium-rich blast furnace slag. Furthermore, this paper explores the enrichment technology combined with microwave fields, which can effectively remove the silicate coating on the surface of perovskite. This enables the effective enrichment of all titanium ions into perovskite in the titanium slag after pretreatment, significantly improving titanium recovery efficiency. This method presents a significant advantage in the future extraction of titanium from titanium-rich blast furnace slag.

Extracting titanium from blast furnace slag still faces challenges. First, the thermodynamic and kinetic models of titanium migration and transformation are incomplete and need further development. Second, high-temperature silicate encapsulation of titanium-bearing phases is difficult to overcome, reducing recovery rates. Third, efficient separation technologies for titanium-rich phases are lacking, leading to suboptimal resource use. Finally, the high reagent consumption in hydrometallurgy raises costs and risks secondary pollution, particularly in the treatment and disposal of acid and alkaline waste, posing environmental concerns.

To improve titanium recovery, and to create an economical and clean treatment process, combining pyrometallurgy and mineral processing with hydrometallurgy in an integrated smelting approach is suggested. Future research should focus on optimizing synergies between process stages, combined with the new mineral processing technology to improve the separation efficiency of the titanium-rich phase and reduce the acid concentration. Additionally, integrating digital technologies and artificial intelligence to optimize workflows, increase automation, and improve intelligent process management will be key trends in future developments.

## Figures and Tables

**Table 1:** Main chemical composition of titanium-bearing blast furnace slag (mass%).

District	TiO_2_	SiO_2_	CaO	MgO	Al_2_O_3_	MFe	TFe	V_2_O_5_	Other
Chengde [28]	12.26	28.30	31.73	11.66	13.42	-	2.52–3.00	0.20	1.28
Yunnan [29]	10.40	25.29	29.14	9.62	11.21	11.79	-	-	2.61
Panzhihua [30]	20.00–24.00	17.00–19.00	20.00–30.00	8.00–8.90	14.00–16.00	1.00–3.00	3.00–4.20	0.20–0.25	0.30–0.50

**Table 2:** Main titanium-bearing minerals and their properties.

Phase	Molecular Formula	Crystal Structure	Density (p/cm)^−3^	Geometry
Perovskite	CaTiO_3_	Cubic Crystal System	4.10	Spindle shaped
Rutile	TiO_2_	Tetragonal Crystal System	4.20–4.30	Tetragonal prismatic, acicular
Anosovite	Ti_3_O_5_	Orthorhombic Crystal System	4.68–4.79	Bundled
Ilmenite	FeTiO_3_	Trigonal Crystal System	4.20–5.20	Tabular, granular
Titanaugite	(Ca,Mg,Ti,Fe,Mn)_2_[(Si,Al,Ti)_2_O]_6_	Monoclinic Crystal System	3.20–3.60	Columnar, tabular
Pseudobrookite	Fe_2_TiO_5_	Orthorhombic Crystal System	4.49	Acicular
Titanium carbonitride	Ti(C,N)	Cubic Crystal System	4.93–5.05	Particulate
Anatase	TiO_2_	Tetragonal Crystal System	3.90	Conical, tabular
Sphene	CaTiSiO_5_	Monoclinic Crystal System	3.50	Wedge shaped, tabular

**Table 3:** Research related to alkali molten salts.

Alkaline Reagent	Calcination Temperature	Acid Leaching Reagent	Impurity Removal Rate	TiO_2_ Recovery	Main Mineral Phases
NaOH(S) [44]	500 °C	50% H_2_SO_4_	Si:45.00%, Al:83.00%	TiO_2_ grade before acid leaching 36.13%, acid dissolution rate 91.21%.	Perovskite
2 mol/L NaOH(L) [45]	95 °C	2.00 mol/L HCl	Si:99.08%, Al:97.30%	97.77%	Rutile
4 mol/L NaOH(L) [46]		4.50 mol/L HCl	Si:99.71%, Al:99.88%	TiO_2_ grade before acid leaching 72.84%, acid digestion rate: 98.61%.	Rutile, Glass phase

**Table 4:** Summary of each method in the hydrometallurgical method.

Reagent	TiO_2_ Grade	Parameter Range	Ti Recovery Effect	Phase
H_2_SO_4_ [18,19,20,21]	17.00–74.00%	Reaction stage (L/S = 1.4–2.2:1; C(H_2_SO_4_) = 70–93%; t = 30 min–40 min); Curing phase (T = 160 °C–250 °C; t = 2–4 h). Leaching phase (C(H_2_SO_4_) = 0–2.5%; T = 50 °C–70 °C; t = 1–8 h)	Leaching rate 80–95%	Perovskite, Silicate, Anosovite, Titanaugite
HCl [41,51,52]	18.40–32.87%	L/S = 7.5–15; C(HCl) > 30%; t = 30 min–2 h; T= 90 °C–120 °C	Leaching rate 75–90%	Perovskite
H_2_SO_4_ + HCl [34]	23.00–40.00%	Cl^−^:SO_4_^2−^ = 1:1; T = 65 °C; t = 10 h; Hydrolysis (T = 160 °C; t = 4 h)	Leaching rate 80%	Perovskite, Titanaugite, Diopside
NaOH [44,45,46]	21.00–79.00%	NaOH:salg = 1.4–4:1; T = 220 °C–350 °C; t = 1.5 h–4 h	Alkaline rate 36.13–72.84%, Leaching rate > 91%	Anosovite, Rutile
(NH_4_)_2_SO_4_ [39,49,53]	18.00–27.00%	(NH_4_)_2_SO_4_: slag = 2–3:1; Pyrolysis: (T = 370 °C–390 °C; t = 1–1.5 h); Leaching(C(H_2_SO_4_) = 5–10%	Leaching rate 85–95%	Perovskite, Diopside, Calcium silicate

**Table 5:** Summary of Research on Metal Reduction Methods.

Researchers	Reducing Agents	Experimental Methods
Zhang [71]	Si	Under an argon atmosphere, Si was mixed with high-titanium slag in a 1:7.3 ratio. The study found that when the reaction temperature exceeded 1773K and the reaction time exceeded 30 min, the formation of Ti_5_Si_3_ was favored, along with better separation from the liquid slag.
Lei [1]		TiO_2_ in TBFS is reduced by Si to form a Si–Ti alloy. Electromagnetic directional solidification is used to separate the Si–Ti alloy into blocky Si and eutectic Si–Ti. Most impurities (Mn, Al, Fe, etc.) can be removed, and high-purity block silicon can be used to prepare higher-purity silicon powder through acid leaching.
Huang [72]	Al	For the preparation of Al–Si–Ti alloys, as the Al content increased to 20%, the Ti recovery rate reached 80%. When the Al content exceeded 24%, the Si recovery rate was 70%. The main phases of the alloy were Ti_5_Si_3_, AlTi_3_, and TiSi_2_.
Lei [73]		By adding 20% CaO and 40% Al, the alloy composition was adjusted to 31.8% Ti, 22.7% Si, 28.5% Al, and 10.2% Fe. After directional solidification and electromagnetic separation, Ti_5_Si_4_, TiSi, Al–Si–Ti, Al–Si–Fe, and Al–Si phases were obtained.

**Table 6:** Beneficiation separation of titanium-containing phase.

Phase	TiO_2_ Grade of Raw Ti Slag	Beneficiation Process	Experimental Methods	Concentrate Indicators
Perovskite [85]	TiO_2_ grade: 19.78%, TiO_2_ grade (Perovskite): 50.84%	Flotation process	Caprylhydroxamic acid: 0.0002 mol/L; PH: 5.50–6.50; Sodium silicate inhibitor: 40.00 mg/L	TiO_2_ grade: 39.44%; TiO_2_ recovery rate: 39.44%
Anosovite [86]	TiO_2_ grade: 36.57%, TiO_2_ grade (Anosovite): 73.20%	Gravity separation	Skimming laps: 5; Feed concentration: 35%; concentrate interception position 3 cm from the inside	TiO_2_ grade: 67.19%; TiO_2_ recovery: 61.62%; anosovite recovery: 68.74%
Rutile [87,88]	33.78%	Gravity separation	Sorting in two stages using a spiral chute, followed by a shaking table test. Another vibration test was performed on the first tailings	TiO_2_ grade: 61.75% (first tailings vibration experiment: TiO_2_ grade 57.50%; rutile recover: 89.22%)
	TiO_2_ grade: 2.57%, TiO_2_ grade (Rutile): 2.40%	Gravity separation–Magnetic separation–Acid washing–Flotation	Collectors: Oil and kerosene; Inhibitor: Na_2_SiF_6_ + Al_2_(SO_4_)_3_ + Na_2_SiO_3_; pH regulator: Na_2_CO_3_	88.25% rutile in concentrate, with 97.80% rutile recovery

## References

[1] Lei Y., Wang C., Ma W.H., Wu J.J., Wei K.X., Li S.Y., Lv G.Q., Morita K. (2019). A novel approach to prepare high-purity Si and Si/TiSi_2_ materials simultaneously using Ti-bearing blast furnace slag. J. Alloys Compd..

[2] Li S.P., Tian L., Zhang Y., Li X., Xie J., Zhang S.B., Liu W.Y., Zhang L. (2024). Determination of antibacterial properties of modified titanium-containing blast furnace slag. Light Met..

[3] Cao L., Zhu K.S., Tang J.J., Wang J., Zhao Y.T., Cheng X.K., Suo X.Z. (2024). Thermodynamic Calculation and Experimental Study on the Recovery of Valuable Metal Elements from Titania-Bearing Blast-Furnace Slag by Silicothermic Reduction. J. Kunming Univ. Sci. Technol..

[4] Lai F.F. (2021). Basic Research on Preparation of Glass-ceramics from Low Ti-bearing Blast Furnace Slag. Master’s Thesis.

[5] Xie S.Y., Jiang W., Wang S.D., Jiang X.X., Zhang D.G., Mao H.C., Zhao F. (2024). Activation Test of Titanium-bearing Blast Furnace Slag by Roasting with Ammonium Chloride. Nonferr. Met. Extr. Metall..

[6] Fan G.Q., Wang M., Dang J., Zhang R., Lv Z.P., He W.C., Lv X.W. (2021). A novel recycling approach for efficient extraction of titanium from high-titanium-bearing blast furnace slag. Waste Manag..

[7] Ghidini T. (2018). Materials for space exploration and settlement. Nat. Mater..

[8] Zhang D.Y., Qiu D., Gibson M.A., Zheng Y.F., Fraser H.L., StJohn D.H., Easton M.A. (2019). Additive manufacturing of ultrafine-grained high-strength titanium alloys. Nature.

[9] Chen Q.Z., Thouas G.A. (2015). Metallic implant biomaterials. Mater. Sci. Eng. R Rep..

[10] Gordon C.P., Engler H., Tragl A.S., Plodinec M., Lunkenbein T., Berkessel A., Teles J.H., Parvulescu A.N., Coperet C. (2020). Efficient epoxidation over dinuclear sites in titanium silicalite-1. Nature.

[11] Ju D.C., Wu Z.Y., Zhang R.L., Wang H.F., Wang F., Yan D.L. (2019). Research progress and prospect on titanium extraction from titanium-bearing BF slag. Mod. Chem. Ind..

[12] Pu Z.H., Jiao H.D., Mi Z.S., Wang M.Y., Jiao S.Q. (2020). Selective extraction of titanium from Ti-bearing slag via the enhanced depolarization effect of liquid copper cathode. J. Energy Chem..

[13] Bo A., Alarco J., Zhu H.Y., Waclawik E., Zhan H.F., Gu Y.T. (2017). Nanojoint Formation between Ceramic Titanate Nanowires and Spot Melting of Metal Nanowires with Electron Beam. ACS Appl. Mater. Interface.

[14] He M.Y., Teng L.M., Gao Y.X., Rohani S., Ren S., Li J.L., Yang J., Liu Q.C., Liu W.Z. (2022). Simultaneous CO_2_ mineral sequestration and rutile beneficiation by using titanium-bearing blast furnace slag: Process description and optimization. Energy.

[15] Nie W.L., Wen S.M., Feng Q.C., Liu D., Zhou Y.W. (2020). Mechanism and kinetics study of sulfuric acid leaching of titanium from titanium-bearing electric furnace slag. Mater. Res. Technol..

[16] Zhou X.J., Zhao H.G., Wang Q., Li N., Wu Z.S. (2016). Leaching behavior of blast-furnace slag with Ti in hydrochloric acid. Light Met..

[17] Zhang R., Dang J., Liu D., Lv Z.P., Fan G.Q., Hu L.W. (2020). Reduction of perovskite-geikielite by methane–hydrogen gas mixture: Thermodynamic analysis and experimental results. Sci. Total Environ..

[18] Wang L., Chen L., Liu W.Z., Zhang G.Q., Tang S.W., Yue H.R., Liang B., Luo D.M. (2022). Recovery of titanium, aluminum, magnesium and separating silicon from titanium-bearing blast furnace slag by sulfuric acid curing-leaching. Int. J. Miner. Metall. Mater..

[19] Li K.M., Wang H.B., Xiao J. (2022). Experimental study on the acidolysis reaction of low-grade titanium slag. Iron Steel Vanadium Titan..

[20] Chen Q.F. (1995). Scale-up Experiment on TiO_2_ and SC_2_O_3_ Recovery from B.F. Slag at Pangang. Iron Steel Vanadium Titan..

[21] Xue X., Li W.B., Wang J.W., Yan S. (2009). Factors Influencing the Acidolysis Ratio of Titanium Extraction from Titanim bearing Blast Furnace Slag. Metal Mine.

[22] Hu M.J., Wei R.R., Hu M.L., Wen L.Y., Ying F.Q. (2018). Nonisothermal carbothermal reduction kinetics of titanium-bearing blast furnace slag. JOM.

[23] Zhang W., Zhang L., Li Y.H., Li X. (2016). Crystallization behavior and growing process of rutile crystals in Ti-bearing blast furnace slag. High Temp. Mater. Process..

[24] Zhang S.Q., Zheng K., Jiang J.X., Zhang S.Y., Xu G. (2021). Effect of operating parameters on high-temperature selective enrichment and precipitation of titanium component in Ti-bearing blast furnace slag and the precipitation mechanism of perovskite. J. Mater. Res. Technol.

[25] Wang J. (2022). Analysis on the separation and extraction technology of titanium components in titanium bearing blast furnace slag. World Nonferrous Met..

[26] Jiang T., Dong H.G., Guo Y.F., Li G.H., Yang Y.B. (2010). Study on leaching Ti from Ti bearing blast furnace slag by sulphuric acid. Miner. Process. Extr. Met..

[27] Smirnov L.A., Koshkarov D.A., Zayakin O.V., Mironov K.V., Krasheninin A.G., Forshev A.A., Kalimulina E.G. (2023). Composition and Properties of TBFSs. Metallurgist.

[28] Tian Y., Chen S.J., Sun Y.Q., Lv Q., Qie Y.N., Liu X.J. (2016). Settlement Process of Iron in Titania Bearing Blast Furnace Slag. Iron Steel Vanadium Titan..

[29] Dong X.H., Tian Y., Su Y. (2024). Study on the preparation of composite adsorbent with titanium containing blast furnace slag and chromium adsorption performance. Chem. Ind. Eng. Prog.

[30] Jian T.F. (2022). Enrichment and Separation of Ti(C,N) During Carbothermal Reduction of Ti-bearing BF Slag. Master’s Thesis.

[31] Chen D.S., Zhao L.S., Liu Y.H., Qi T., Wang J.C., Wang L.N. (2013). A novel process for recovery of iron, titanium, and vanadium from titanomagnetite concentrates: NaOH molten salt roasting and water leaching processes. J. Hazard. Mater..

[32] Sun H.J., Zhou G.B., Peng T.J., Zhou F., Wu X., He S.Q. (2015). Recove by of Titanium from Titanium-Rich Product Prepared from High Ti-Bearing Blast Furnace Slag by Sulfuric Acid Leaching. Min. Metall..

[33] Liu Y., Chen X.G., Mao S.D., Xiao Y.D., Li J.C. (2024). Extraction of Valuable Metals from Titanium-bearing Blast Furnace Slag by Acid Leaching. J. Wuhan Univ. Technol.-Mater. Sci. Ed..

[34] Tian M., Liu Y.H., Zhao W., Wang L.N., Chen D.S., Zhao H.X., Meng F.C., Zhen Y.L., Qi T. (2022). Preparing Metatitanic Acid from Perovskite-Type Titanium Slag Using a Sulfuric-Chloric Mixture Acid. JOM.

[35] Xiong Y., Li C., Liang B., Xie J. (2008). Leaching behavior of air cooled Ti-bearing blast-furnace slag in hydrochloric acid. Chin. J. Nonferr. Met..

[36] Xiong Y., Li C., Liang B., Xie J. (2008). Extraction and separation of titanium from air-cooled Ti-bearing blast furnace slag. Chin. J. Process Eng..

[37] Zhu X.F., Zheng S.L., Zhang Y., Fang Z.G., Zhang M., Sun P., Li Q., Zhang Y., Li P., Jin W. (2019). Potentially more ecofriendly chemical pathway for production of high-purity TiO_2_ from titanium slag. ACS Sustain. Chem. Eng..

[38] Deng Y., Zheng C.L., Li J.G., Zeng Y.N., Ma T. (2022). Titanium enrichment process of titanoum bearing blast furnace slag and untilization of titanium resources. China Metall..

[39] Xiong Y.J., Aldahri T., Liu W.Z., Chu G.R., Zhang G.Q., Luo D.M., Yue H.R., Liang B., Li C. (2020). Simultaneous preparation of TiO_2_ and ammonium alum, and microporous SiO_2_ during the mineral carbonation of titanium-bearing blast furnace slag. Chin. J. Chem. Eng..

[40] Gong Y.C., Qiu K.H., Li J.H., Li J.F. (2010). Preparation of Titanium-rich Raw Material from Titanium-bearing Blast Furnace Slag by Low Temperature Acid-Base Method. Min. Metall. Eng.

[41] Huang X.L., Zhong S., Tang S.Y., Song L., Li H.J., Liang B. (2023). Production of high-purity rutile titanium dioxide by leaching water-quenched titanium-bearing blast furnace slag with hydrochloric acid. Iron Steel Vanadium Titan..

[42] Li C., Qiu K.H., Zhang P.C., Cao L. (2011). Separation of the Main Components from Ti-bearing Blast Furnace Slag by Mixed Acid Leaching Method. Chin. J. Process Eng..

[43] Sun K., Wu J.H., Ma Y.Y., Hu Z.W., Wu H.B., Chen H.Q., Yu X., Qin W., Li Z. (2000). Fundamental study of new treatment process for titaniferous blast furnace slag at pangang using phase separation. Iron Steel Vanadium Titan..

[44] He S.Q., Peng T.J., Sun H.J. (2019). Titanium recovery from Ti-bearing blast furnace slag by alkali calcination and acidolysis. JOM.

[45] Han J.Q., Zhang J., Zhang J.H., Chen X., Zhang L., Tu G.F. (2021). Extraction of vanadium and enrichment of titanium from modified Ti-bearing blast furnace slag. Hydrometallurgy.

[46] Han J.Q., Zhang J., Zhang J.H., Chen X., Zhang L., Tu G.F. (2022). Recovery of Fe, V, and Ti in modified Ti-bearing blast furnace slag. Trans. Nonferr. Met. Soc. China.

[47] Nduagu E.I., Highfield J., Chen J., Zevenhoven R. (2014). Mechanisms of serpentine–ammonium sulfate reactions: Towards higher efficiencies in flux recovery and Mg extraction for CO_2_ mineral sequestration. RSC Adv..

[48] Mohamed S., Merwe E.M., Altermann W., Doucet F. (2016). Process development for elemental recovery from PGM tailings by thermochemical treatment: Preliminary major element extraction studies using ammonium sulphate as extracting agent. Waste Manag..

[49] Bian Z.Z., Feng Y.L., Li H.R. (2020). Extraction of valuable metals from Ti-bearing blast furnace slag using ammonium sulfate pressurized pyrolysis–acid leaching processes. Trans. Nonferr. Met. Soc. China.

[50] Zhang Y. (2014). Recovery of titanium from titanium bearing blast furnace slag by sulphate melting. Can. Metall. Q..

[51] Cao H.Y., Fu N.X., Kang C.B., Zhang L., Yang J., Sui Z.Y., Feng N.X. (2008). Pressure leaching of the modified Ti-bearing blast furnace slag by hydrochloric acid. Multipurp. Util. Miner. Resour..

[52] Zhang P., Liu D.J., Mao X.H. (2013). Study on preparation of TiO_2_ from aqueous TiCl_4_ solution by pyrohydrolysis. Iron Steel Vanadium Titan..

[53] Wang L., Liu W.Z., Hu J.P., Liu Q., Yue H.R., Liang B., Zhang G.Q., Luo D.M., Xie H.P., Li C. (2018). Indirect mineral carbonation of titanium-bearing blast furnace slag coupled with recovery of TiO_2_ and Al_2_O_3_. Chin. J. Chem. Eng..

[54] Zhen Y.L., Zhang G.H., Chou K.C. (2016). Mechanism and kinetics of the carbothermic reduction of titanium-bearing blast furnace slag. Metall. Res. Technol..

[55] Adipuri A., Li Y., Zhang G.Q., Ostrocski O. (2011). Chlorination of reduced ilmenite concentrates and synthetic rutile. Int. J. Miner. Process..

[56] Jiang T., Xue X.X., Duan P.N., Liu X., Zhang S.H., Liu R. (2008). Carbothermal reduction-nitridation of titania-bearing blast furnace slag. Ceram. Int..

[57] Zhang G.H., Gou H.P., Wu K.H., Chou K.C. (2017). Carbothermic reduction of Panzhihua ilmenite in vacuum. Vacuum.

[58] Zhang F.Q., Guo Y.F., Qiu G.Z., Chen F., Wang S., Sui Y.L., Jiang T., Yang L.Z. (2018). A novel process for preparation of titanium dioxidefrom Ti-bearing electric furnace slag: NH_4_HF_2_−HF leachingand hydrolyzing process. J. Hazard. Mater..

[59] Fan G.Q., Dang J., Zhang R., Lv Z.P. (2020). Synthesis of Ti(C, O, N) from ilmenite at low temperature by a novel reducing and carbonitriding approach. Int. J. Energy Res.

[60] Jiao H.D., Tian D.H., Tu J.G., Jiao S.Q. (2018). Production of Ti–Fe alloys via molten oxide electrolysis at a liquid iron cathode. RSC Adv..

[61] Zhou X.L., Lu X.G. (2010). Preparation of titanium alloy by direct reduction of Ti-bearing blast furnace slag. Chin. J. Nonferr. Met..

[62] Martin-Treceno S., Weaver N., Allanore A., Bishop C.M., Marshall A.T., Watson M.J. (2020). Electrochemical behaviour of titanium-bearing slag relevant for molten oxide electrolysis. Electrochim. Acta.

[63] Liang X.X., Weng W., Gu D., Xiao W. (2019). Nickel based oxide film formed in molten salts for efficient electrocatalytic oxygen evolution. J. Mater. Chem. A.

[64] Weng W., Zeng C., Xiao W. (2019). In Situ Pyrolysis Concerted Formation of Si/C Hybrids during Molten Salt Electrolysis of SiO_2_@Polydopamine. ACS Appl. Mater. Interfaces.

[65] Du Y. (2022). Mineralphase Transformation and Selective Separation of Titanium in Titaniumbearing Slagvia Super-Gravity. Ph.D. Thesis.

[66] Li J.C., Guo Z.C., Gao J. (2014). Isothermal enriching perovskite phase from CaO–TiO_2_–SiO_2_–Al_2_O_3_–MgO melt by super gravity. ISIJ Int..

[67] Gao J., Zhong Y.W., Guo Z.C. (2016). Selective separation of perovskite (CaTiO_3_) from titanium bearing slag melt by super gravity. ISIJ Int..

[68] Zhang C., Yang F., Chen C.G., Guo Z.M. (2021). The microstructure and mechanical properties of extra low interstitials (ELI) Ti-6Al-4V alloys manufactured from hydrideedehydride (HDH) powder. J. Alloys Compd..

[69] Dias Corpa Tardelli J., Bolfarini C., Candido Dos Reis A. (2020). Comparative analysis of corrosion resistance between beta titanium and Ti-6Al-4V alloys: A systematic review. J. Trace Elem. Med. Biol..

[70] Feng C., Chu M.S., Tang J., Tang Y.T., Liu Z.G. (2016). Effect of CaO/SiO_2_ and Al_2_O_3_ on viscous behaviors of the titanium-bearing blast furnace slag. Steel Res. Int..

[71] Zhang G.H., Wang K.F. (2018). Preparation of Ti_5_Si_3_ by silicothermic reduction of titanium-bearing blast furnace slag. Can. Metall. Q..

[72] Huang Q.Y., Lv X.W., Huang R., Song J.J. (2013). Preparation of Ti-Si-Al alloy by aluminothermic reduction of TiO_2_ bearing blast furnace slag. Can. Metall. Q..

[73] Lei Y., Sun L.E., Ma W.H., Ma X.D., Wu J.J., Li S.Y., Morita K. (2018). An approach to employ titanium-bearing blast-furnace slag to prepare Ti and Al-Si alloys. J. Alloys Compd..

[74] Liu S.S., Guo Y.T., Qiu G.Z., Jiao T., Chen F. (2013). Preparation of Ti-rich material from titanium slag by activation roasting followed by acid leaching. Trans. Nonferr. Met. Soc. China.

[75] Safdar F., Zhang Y., Zheng S.L., Chen X., Sun P., Zhang Y., Li P. (2020). Recovery of TiO_2_-enriched material from vanadium titano-magnetite concentrates by partial carbon reduction and mild acid leaching. Hydrometallurgy.

[76] Fu N.X., Zhang Y.W., Sui Z.T. (1997). Influence of chemical composition on the precipitation behaviour of chalcogenide phases from high titanium blast furnace slag. Min. Metall. Eng..

[77] Zhao J., Shi X.F., Zhao K., Yu H. (2024). Effect of Binary Alkalinity on Viscosity Temperature Characteristics and Crystallization Process of High Titanium Slag. Hebei Metall..

[78] Du Y., Gao J.T., Lan X., Guo Z.C. (2020). Recovery of rutile from Ti-Bearing blast furnace slag through phase transformation and super-gravity separation for dielectric material. Ceram. Int..

[79] Shi J.J., Chen M., Santoso I., Sun L.F., Jiang M.F., Taskinen P., Jokilaakso A. (2022). 1250 °C liquidus for the CaO-MgO-SiO_2_-Al_2_O_3_-TiO_2_ system in air. Ceram. Int..

[80] Xie J., Ye Q., Yang F., Qian G.M., Zhang H.J. (2023). Selective enrichment and extraction of well-catalyzed perovskites on melting-microwave heating process from blast furnace slag. J. Clean. Prod..

[81] Ren S., Zhang J.L., Xing X.D., Su B.X., Wang Z., Yan B.J. (2014). Effect of B_2_O_3_ on phase compositions of high Ti bearing titanomagnetite sinter. Ironmak. Steelmak..

[82] Li Z.M., Sun Y.Q., Liu L.L., Wang X.D., Zhang Z.T. (2015). Enhancement of rutile formation by ZrO_2_ addition in Ti-bearing blast furnace slags. ISIJ Int..

[83] Fan H.L., Wang R.X., Xu Z.F., Duan H.M., Chen D.F. (2021). The effect of B_2_O_3_ on the structure and properties of titanium slag melt by molecular dynamics simulations. J. Mater. Res. Technol..

[84] Du Y., Gao J.T., Lan X., Guo Z.C. (2018). Selective precipitation and in situ separation of rutile crystals from titanium bearing slag melt in a super-gravity field. CrystEngComm.

[85] Zhang S.Q., Wang W.Q., Zheng Y., Ren P.K., Yan W., Deng J. (2017). Study on the Flotation Separation of Modified Ti-bearing Blast Furnace Slag. Iron Steel Vanadium Titan..

[86] Ji S., He Q., Li Q.J., Zheng S.B. (2018). Research on Gravity Separation of Anosovite from Ti-bearing Slag. Iron Steel Vanadium Titan..

[87] Wang Z.X., Zheng Y.X., Huang X., Wang X.D., Peng J.L., Dai Z. (2024). Gravity Separation Tests of a Complex Rutile Ore. Minerals.

[88] Shi G.M., Zhou Y.C., Li M. (2018). Beneficiation Experiment of a Refractory and Low Grade Fine Grain Rutile Ore in Henan Province. Gold Sci. Technol..

